# Attenuation of Cortically Evoked Motor-Neuron Potential in Streptozotocin-Induced Diabetic Rats: A Study about the Effect of Diabetes upon Cortical-Initiated Movement

**DOI:** 10.1155/2020/1942534

**Published:** 2020-02-25

**Authors:** Jesper Guldsmed Madsen, Jakob Appel Østergaard, Henning Andersen, Michael Pedersen

**Affiliations:** ^1^Comparativ Medicine Lab, Aarhus University Hospital, Aarhus, Denmark; ^2^Department of Endocrinology and Diabetes, Aarhus University Hospital, Aarhus, Denmark; ^3^Steno Diabetes Center Aarhus, Aarhus University Hospital, Aarhus, Denmark; ^4^Department of Neurology, Aarhus University Hospital, Aarhus, Denmark

## Abstract

*Aims/Hypothesis*. The complications affecting the peripheral nervous system, associated with diabetes mellitus, have been the focus of considerable research. Comparably less research has focused upon the effect of diabetes upon the central nervous system. In this study, we investigate the effect of diabetes upon motor-neuron potentials evoked in the motor cortex of streptozotocin diabetic rats.

**Methods:**

In this study, we investigated the cortical-evoked motor-neuron potentials in streptozotocin-induced diabetic rats. Cortical potentials were evoked using direct current stimulation to the motor cortex, and the resulting evoked potentials were recorded in the sciatic nerve. As voluntary movement consists of repeated activation of muscles, repeated stimulation trials were used to determine the effect of diabetes upon the animals' ability to recuperate between stimulations.

**Results:**

Our findings showed that diabetes severely decreased the amplitude of cortical-evoked potentials and compromised the recuperation of motor neurons between activation. *Conclusion/Interpretation*. The reduced amplitude and weakened recuperation of diabetic motor neurons potentially may contribute to impaired transmission in motor pathways and thereby motor dysfunction.

## 1. Introduction

Diabetic neuropathy (DN) consists of a complex array of symptoms caused by slow degeneration of peripheral nerves [1], and the development of DN depends on the amount and type of affected peripheral nerves. DN is usually associated with loss of sensation or hyperalgesia in sensory nerves [2], whereas DN in autonomic nerves can lead to autonomic dysregulation of the heart and the gastrointestinal tract [3]. Depending on the site of interest, DN is evaluated clinically by several methods. Sensory tests include thermal, vibration, and monofilament tests (Semmes-Weinstein test) [4, 5]. Electrophysiology allows measurements of the nerve conduction speed and amplitude of nerve signals in both sensory and motor neurons [6, 7], and decreased conduction velocity is often demonstrated in the developing DN, including demyelination, Schwann cell damage, and axonal degeneration [8, 9]. These complications are often accompanied by reduction in C-fiber function [10] with secondary pain and loss of sensation.

However, these methods are inadequate for evaluation of the impact of diabetes upon voluntary movement. Thus, we aimed to investigate the effect of diabetes on peripheral motor-neuron potential by mimicking cortical-evoked movement of a limb. We hypothesize that diabetes will diminish cortical-evoked motor-neuron potentials, which could be detrimental for both muscle strength and coordination. As voluntary movement consists of repeated activation of skeletal muscles, we also examined the effect of continued cortical activation of a peripheral motor neuron. Studies in diabetic neurons have shown higher degrees of attenuation of nerve potentials when stimulation occurred with high frequencies compared to those acquired in healthy subjects [11, 12]. In this study, we studied the possible attenuation at lower frequencies, which mimic normal limb movement.

## 2. Materials and Methods

Female Wistar rats (Janvier, Saint-Berthevin Cedex, France) were divided into a diabetic group (*n* = 6) and a nondiabetic group (*n* = 6). Female rats were used, as they tend to display less hierarchy and are thus easier to keep stabled. Furthermore, our lab has developed successful protocols for female rats [13, 14]. Diabetes was induced by streptozotocin (STZ; Sigma-Aldrich, Copenhagen, Denmark) as previously described [15]. In brief, eight-week-old female Wistar rats were injected with a single dose of STZ (55 mg/kg body weight, dissolved in cold 0.01 M, pH 4.5 sodium citrate buffer after an overnight fast). Drinking water was supplemented with 15 g/L of sucrose for 48 h following STZ administration. Following this supplementation, free access to food and water was maintained for 14 days to allow DN to develop [16, 17].

Electrophysiological experiments were performed in 10-week-old rats (250–400 g). Rats were anesthetized using ip administration of ketamine (100 mg/kg) and xylazine (10 mg/kg) [18]. Surgical access to the sciatic nerve was obtained via the groin. Stainless steel stimulation electrodes were fastened subcutaneously to the animal's scalp, 1 cm caudal of the eyes, and a platinum recording electrode was placed on the exposed sciatic nerve. Stimulation was supplied using a signal generator (WPI model A310; World Precision Instruments, Sarasota, FL, USA) through a signal isolator with 100% current amplitude (WPI model A365). The stimuli consisted of two pulses of 10 V with 100 *μ*s duration and 100 *μ*s intervals [19]. The stimulation rate (SR) varied between 0.1 Hz and 1 Hz in each animal. The animals were subjected to two stimulation series, and each series consisted of 10 stimulations with an SR of either 0.1 Hz or 1 Hz. These SRs fall well within the range of repetitive muscle activation speeds in rats, such as during walking or running, and should thus mimic voluntary repetitive activation of the muscles. Each stimulation series was applied once in each animal with a minimum of 2 min recovery time between series.

**Figure 1:**
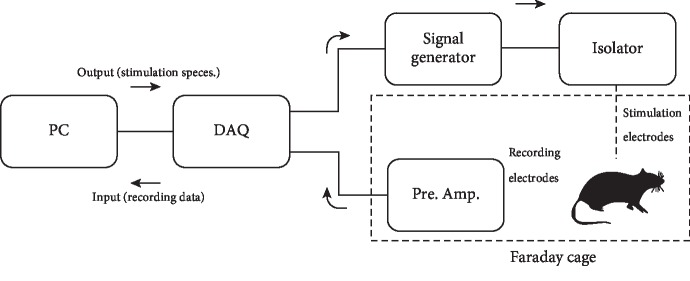
Diagram of the electrophysiological experimental circuit. A data acquisition unit (DAQ) converts the digital output from a PC to an analogue signal which triggers a signal generator to produce a stimulus pulse of the desired duration and voltage. This pulse is passed through an isolator; this enables the current to be increased if needed and isolates the recording part of the circuit from the power supply, reducing noise. The stimulus pulse is led through stainless steel electrodes into the experimental animal. Platinum recording electrodes record the resulting stimulus response which is amplified and filtered through a preamplifier. The DAQ then converts the analogue stimulus response to a digital signal which is stored on the PC. The recordings were carried out inside a Faraday cage.

Stimulation was controlled by a PC, using LabVIEW software (National Instruments, Austin, TX, USA), connected to the signal generator via a digital-to-analog converter (NI USB 6152; National Instruments). Evoked responses were recorded via the recording electrode, through a preamplifier (Grass P50; Grass, West Warwick, RI, USA) that was set to 100x amplification and a bandpass filter in the range of 0.1-3 kHz. The sampling rate was 20 kHz. Compound action potentials in the sciatic nerves were recorded in response to depolarization of the motor cortex, and the amplified recordings were digitally stored via the NI USB 6152 digital-to-analog converter. All recordings were carried out inside a Faraday cage, and the preamplifier was grounded via the Faraday cage to a common earth. A schematic of the experimental setup is shown in Figure 1.

Peak-to-peak amplitudes of the evoked compound action potentials were used to quantify the impact of diabetes upon the pathway used for voluntary movement, from the motor cortex, through the spinal cord, and finally in a peripheral motor nerve. The peak-to-peak amplitude was calculated by subtracting the lowest recorded voltage of the evoked compound action potentials from the highest recorded voltage.

In order to ensure that stimulations were supramaximal, a series of current ramp tests were conducted on 6 additional animals not included in the tests described above. Using the procedure described above, 6 nondiabetic rats (250-400 g) were subjected to stimulations, first at 50%, then 80%, and finally 100% current output, with a minimum 2 min recovery time between stimulations. Response levels at the different current outputs were then compared to establish if increased current output had any effect upon the responses measured in the sciatic nerve.

Experimental permission was granted by the national council for animal research (#2015-15-0201-00719).

### 2.1. Statistics

Statistical analysis was carried out using MATLAB software (MathWorks, Natick, MS, USA). Peak-to-peak amplitude data was pooled to correspond to the variables: (i) stimulation rate and (ii) whether the data was gathered from diabetic or control animals. Once pooled, multiway ANOVA (*N*-way ANOVA) was performed upon the data vectors to investigate whether the stimulation rate and/or diabetes had an effect upon the means of the vectors. Ramp test data was pooled corresponding to current output. One-way ANOVA was then performed to investigate whether changes in the current output had a significant effect upon response amplitudes. *p* values below 0.05 indicated a significant effect of the variable in question. All data are available (doi:10.6084/m9.figshare.4542439.v1). This study received no external funding.

## 3. Results

**Figure 2:**
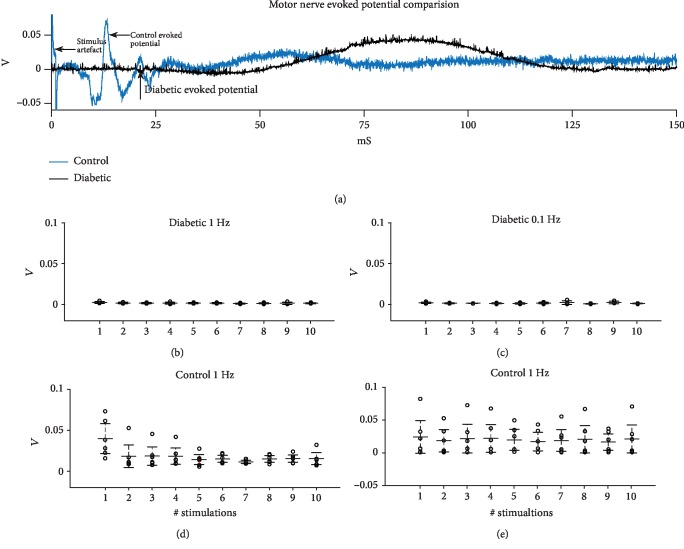
Motor nerve evoked potential comparison. (a) Comparison of health control and diabetic motor nerve evoked potential. (b) Absolut peak-to-peak amplitude of diabetic animal-evoked potentials at a 1 Hz stimulation rate. (c) Absolut peak-to-peak amplitude of diabetic animal evoked potentials at a 0.1 Hz stimulation rate. (d) Absolut peak-to-peak amplitudes of control animal evoked potentials at a 1 Hz stimulation rate. (e) Absolut peak-to-peak amplitudes of control animal evoked potentials at a 0.1 Hz stimulation rate. Bars indicate means and 95% confidence intervals.

Figure 2(a) shows a comparison of evoked potentials in a diabetic and a control animal. The peaks shown in Figure 2(a) are the compound action potentials of the motor neurons of the sciatic nerve. Several peaks are visible in the control animal; this is likely caused by the fact that a twin pulse stimulation is used. As such, some evoked potentials will arrive at the recording electrode before others and produce a more complex wave form. Figures 1(b)–1(e) show the peak-to-peak amplitudes of diabetic (*n* = 6) and control animals (*n* = 6), evoked at 1 and 0.1 Hz. Lines indicate mean values and 95% confidence intervals of peak-to-peak amplitudes for the 10 consecutive stimulations. Note that there is an order of magnitude difference between the response amplitude in diabetic and control animals. Statistical analyses with ANOVA test demonstrated an interaction between the disease state (diabetes vs. nondiabetes) and the peak-to-peak amplitude of evoked potentials, i.e., peak-to-peak amplitude differed between diabetic and nondiabetic animals (*p* < 0.01). SR was not found to have any significant effect upon peak-to-peak amplitudes in either healthy controls or diabetic animals.

**Figure 3:**
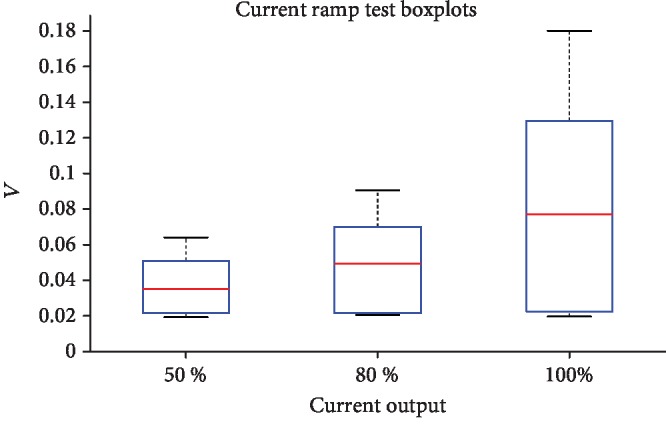
Boxplots of ramp test data at the different current levels. Red lines indicate median peak-peak amplitude of evoked potential responses. Boxes represent 25^th^ and 75^th^ percentiles and whiskers indicate the full extent of the data for each current level.

Results from the ramp test are shown in Figure 3. Here, boxplots of the peak-peak amplitudes of the evoked potentials at different stimulation currents are displayed. Despite that medial evoked potential amplitude increased with increasing current, statistical analysis with ANOVA tests did not demonstrate a significant effect of increasing current output from 50%, through 80% to 100% (*p* = 0.159). This indicates that our stimulations were supramaximal, i.e., the motor cortices stimulated at 100% current output were responding at maximum levels.

## 4. Discussion

The purpose of this study was to investigate the cortical-evoked motor-neuron potentials in response to repeated stimulations, and measurements in the sciatic nerve were conducted in both diabetic and control rats. We found that the diabetic rats generated motor potentials with lower amplitude as compared to control rats. We did not find, however, that the healthy or the diabetic group had a reduced capacity to maintain potential amplitudes during continued stimulations. These results suggest an effect of diabetes per se irrespective of the presence of neuropathy. The reduced amplitude of the evoked potentials in the diabetic group found in this study could contribute to the decreased muscle strength observed in studies of the effects of diabetes on muscle strength in patients with DN [20–22] and decreased isometric muscle strength in response to hyperglycemia [23]. Studies have shown that diabetes has effects upon muscle activation and coordination in the lower limbs, and this study aimed at investigating whether higher attenuation, in response to continued stimulation, in diabetic nerves could explain these effects [24, 25]. However, this study did not find any evidence of attenuation in either healthy or diabetic animals at the stimulation rates used. As mentioned, studies have found higher degrees of attenuation in diabetic nerves in response to higher frequency stimulation [11, 12], but in order to investigate possible effects of diabetes upon voluntary movement, slower frequencies, closer to those experienced during normal movement, such as walking, were used.

At first, DN affects the most distal parts of the peripheral nervous system [26], in part caused by the well-established length dependency of DN in peripheral nerves [27, 28]. Consequently, the choice of nerve and the exact site of recordings influenced the measured amplitudes. In this study, electrophysiological recordings were carried out in the sciatic nerve, but if DN was present, it would appear more severe if more distal nerves were employed.

This study found a significant effect of STZ upon the centrally evoked motor potentials; however, the method used here does not eliminate the possibility that the effects observed are caused by direct neurotoxicity by STZ upon the peripheral nerves. STZ is a glucose analogue and is selectively taken up by the GLUT-2 transporter, causing *β*-cell death and subsequent hyperglycemia. As such, a direct toxic effect upon the nerves seems unlikely. A study has been dedicated to this issue, which did not find any direct toxicity of STZ in rodents, irrespective of hyperglycemia [29].

STZ is also associated with muscle atrophy in diabetic rodent models. While it is possible that muscle atrophy occurred in the animals used in this study, recordings of motor nerve potentials were done directly from the nerves themselves, not from the muscle. Therefore, possible muscle atrophy in the animals should not have influenced the results.

## 5. Conclusion

This study was designed to investigate the cumulative effect of diabetes on the entire pathway, from potentials evoked in the motor cortex to recorded potentials in a peripheral motor neuron. These effects may be caused by reduced excitability of the motor cortex or potentially by poor synaptic transmission between motor neurons in the spinal cord. Finally, it may be explained by a compromised function of the peripheral motor neurons themselves, as found in DN. We found a clear effect of diabetes on cortically evoked potentials in STZ-induced diabetic rats. Amplitude of evoked potentials was negatively affected in the diabetic group. Further investigation is needed to identify the exact location(s) of this effect.

## Data Availability

The data used to support the findings of this study are available from the corresponding author upon request.
